# Photobiomodulation for radiotherapy-induced dysgeusia in head and neck cancer patients: a pilot randomized clinical trial

**DOI:** 10.1007/s10103-026-05090-1

**Published:** 2026-11-05

**Authors:** Priscilla Parente Viana, Flávia Carvalho Pinto, Victor Bento Oliveira, Hanna Emilly Lima Batista, Vitória Isterfany Pimenta Silva, Denise Hélen Imaculada Pereira de Oliveira, Filipe Nobre Chaves, Marcelo Bonifácio da Silva Sampieri

**Affiliations:** 1 Universidade Federal do Ceará, Sobral, Brazil; 2 Universidade Federal de Minas Gerais, Belo Horizonte, Brazil

**Keywords:** Head and neck neoplasms, Radiotherapy, Dysgeusia, Photobiomodulation

## Abstract

Dysgeusia is a frequent complication in patients with head and neck cancer (HNC) undergoing radiotherapy and may negatively affect nutritional status and quality of life. This pilot study evaluated the effect of photobiomodulation (PBM) on the management of radiotherapy-induced dysgeusia. Sixteen patients undergoing radiotherapy for HNC were randomly assigned to either a PBM group (n = 8) or a zinc sulfate group (n = 8). Objective taste identification tests evaluating sweet, bitter, sour, and salty tastes were performed at three time points: before radiotherapy (T0), at the 15th radiotherapy session (T1), and at the 30th radiotherapy session (T2). Salty taste was the most affected taste modality in both groups. At T2, sweet taste was correctly identified by 75.0% of patients in the zinc group and 50.0% in the PBM group. In contrast, bitter taste was preserved in 87.5% of patients in the PBM group and 75.0% in the zinc group. Sour taste was preserved in 87.5% and 62.5% of patients in the PBM and zinc groups, respectively, whereas salty taste was preserved in 50.0% and 25.0% of patients, respectively. No statistically significant differences were observed between groups at T0, T1, or T2 (all p > 0.05). Although no statistically significant differences were identified, PBM was associated with better preservation of sour, bitter, and salty taste perception, particularly after the 30th radiotherapy session. These preliminary findings suggest that PBM may represent a promising supportive strategy for the management of treatment-related dysgeusia in patients with HNC undergoing radiotherapy.

## Introduction

With approximately 41,000 new cases annually, head and neck cancer (HNC) represents 3.6% of all malignant tumors in humans. The majority of patients are male (80%), under 50 years of age, have low levels of education, and consume alcohol and tobacco. HNC largely affects the oral cavity and larynx, and is frequently diagnosed at an advanced stage (70–80%). This directly impacts the effectiveness of treatments, quality of life, and patient survival [1]. HNC is primarily treated with surgery in combination with radiotherapy and/or chemotherapy. Conventional head and neck radiotherapy usually involves high doses of radiation (60 Gy or more) in fractionated daily doses, and in accordance with the diagnosis and clinical stage. Acute and chronic side effects can occur due to the involvement of various tissues in the radiation field. The severity of these complications depends on multiple factors, such as the volume of irradiated tissue, radiation dose, and individual patient characteristics including smoking, alcoholism, poor oral hygiene, professional dental care, and immune system health [2, 3].

Antineoplastic treatment in the head and neck region has several side effects, which can be debilitating and significantly affect both quality of life and patient prognosis [4, 5]. The most common side effects are oral mucositis, xerostomia, candidiasis, dysphagia, hyposalivation, dysgeusia, edema, radiation caries, radiodermatitis, and trismus [2, 4, 6]. These complications are associated with morbidity, mortality and increased healthcare costs. They also tend to compromise patient adherence to cancer therapy protocols, leading to unsatisfactory outcomes [7, 8].

Dysgeusia is a type of taste disorder defined as a distortion in the perception of food flavor [9]. It is a complex phenomenon and includes difficulties related to smell. It results in reduced interest in food and in the quality of life. It can be responsible for energy and nutritional deficiencies, as well as complications such as weight loss [10, 11]. After radiotherapy the prevalence of dysgeusia is estimated at 66.5%. After chemo-radiotherapy the prevalence is estimated at 76.0%. Approximately 15% of patients continue to experience dysgeusia after the end of treatment [12, 13]. The incidence and severity of taste alterations depend on the area being irradiated during treatment and the cumulative radiation dose [14].

Dysgeusia may be associated with zinc deficiency, salivary dysfunction, and alterations in the central nervous system. It has also been linked to the use of medications, including antihypertensive agents, antibiotics, and chlorhexidine, as well as to infections, mucositis, hyposalivation, poor oral hygiene, and chemotherapy [9]. However, in patients undergoing radiotherapy for HNC, taste dysfunction is primarily associated with radiation-induced damage to the gustatory system [15]. Symptoms often appear at the second or third week and may persist for several weeks or even months. The taste buds as well as the receptors of the nasal epithelium are radiosensitive, leading to the degeneration of their histological architecture [7, 18]. Radiotherapy may disrupt taste bud structure, impair the renewal of taste receptor cells, and cause direct damage to differentiated gustatory cells. In particular, suppression of basal cells may reduce the number of type II taste receptor cells, which are involved in the detection of sweet, bitter, and umami stimuli [15]. Additionally, salivary flow is reduced, revealing biochemical alteration and increased viscosity which creates a mechanical barrier that hinders physical contact between the tongue and food [7, 8].

Regarding dysgeusia, a factor that must be considered is that modern radiation techniques, such as intensity-modulated radiotherapy (IMRT) and helical IMRT, have improved the ability to spare healthy tissues, including salivary glands and oral structures, reducing radiation-related toxicities. However, despite these advances, taste dysfunction remains a frequent complication among patients undergoing treatment for HNC [17].

The treatment of dysgeusia includes nutritional care, rinsing with bicarbonate water, frequent fluid intake, good oral hygiene, saliva substitutes, treatments related to xerostomia, and zinc sulfate [16, 18]. However, the effectiveness of zinc sulfate in the treatment of dysgeusia is debated [9]. Dysgeusia is clinically assessed by measuring the detection or recognition thresholds for the five basic tastes: sweet, bitter, sour, salt, and umami (the characteristic taste of protein-rich foods) using filter paper strips impregnated with different concentrations of tastants or, in laboratory settings, an electrogustometer. Qualitative alterations are reported through patient complaints, interviews, and in clinical observations [11]. Furthermore, no universally accepted preventive or therapeutic protocol has been established for treatment-related dysgeusia.

Photobiomodulation therapy (PBM) has recently shown significant potential in preventing and/or reducing the effects of dysgeusia [6]. Photobiomodulation is a form of phototherapy. Visible, red, and infrared light is absorbed by endogenous chromophores, triggering biological reactions [19]. Recent studies suggest that the mechanism of action of photobiomodulation is related to action on cytochrome c oxidase (CcO) in the mitochondrial respiratory chain (facilitating electron transport) [20]. This results in an increase in the transmembrane proton gradient, which drives the production of adenosine triphosphate (ATP), and an increase in bioavailability to fuel the functions of cellular metabolism [20]. PBM has demonstrated anti-inflammatory effects, modulation of oxidative stress, and stimulation of tissue repair processes [6, 21]. Considering that there are no treatment protocols for chemotherapy/radiotherapy-induced dysgeusia and that photobiomodulation (PBM) is a low-cost therapeutic modality, this pilot randomized clinical trial study aimed to evaluate the efficacy of PBM therapy in reducing dysgeusia in patients undergoing radiotherapy for HNC treatment.

## Methods

### Study design

A pilot randomized clinical trial study was conducted to evaluate the efficacy of PBM in treating treatment-related dysgeusia in patiens undergoing radiotherapy for HNC. The research was carried out at the Santa Casa de Misericórdia de Sobral, in collaboration with the Stomatology outpatient clinic of the Federal University of Ceará (UFC) - Sobral *Campus*, during the year 2025.

### Inclusion and exclusion criteria

The sample consisted of all patients who were scheduled to undergo radiotherapy for HNC. Patients were excluded if they: (1) abandoned the treatment or the study; (2) did not commit to attending all stages of the research; (3) did not sign the informed consent form; (4) had undergone previous head and neck radiotherapy; (5) were undergoing palliative treatment. Patients aged 18–90 years who were referred for radiotherapy as part of their head and neck cancer treatment, and signed the informed consent form, were eligible for participation regardless of: primary tumor site; tumor stage; concomitant chemotherapy; previous surgery; and radiation dose.

All patients who met the inclusion criteria were fully informed about the characteristics of the study and invited to participate. Those who accepted were assessed for demographic data, including sex, age, habits, tobacco use, and alcohol consumption. In addition, clinical variables were collected, including tumor type, location, and stage (8th edition of the AJCC staging system), TNM classification, antineoplastic treatment protocol, prescribed radiation dose, and the use of concomitant chemotherapy. They were provided with an oral care protocol, including dental treatment when necessary (scaling, filling, extraction), with instructions on oral care during antineoplastic treatment.

### Outcomes

#### Primary outcome

The primary outcome was the difference between the photobiomodulation and zinc sulfate groups in the proportion of patients who correctly identified each taste modality at baseline (T0), the 15th radiotherapy session (T1), and the 30th radiotherapy session (T2).

#### Secondary outcome

Secondary outcomes included within-group changes in taste identification over time and changes in the proportion of patients correctly identifying each individual taste modality between T0, T1, and T2.

### Randomized clinical trial

**Fig. 1:**
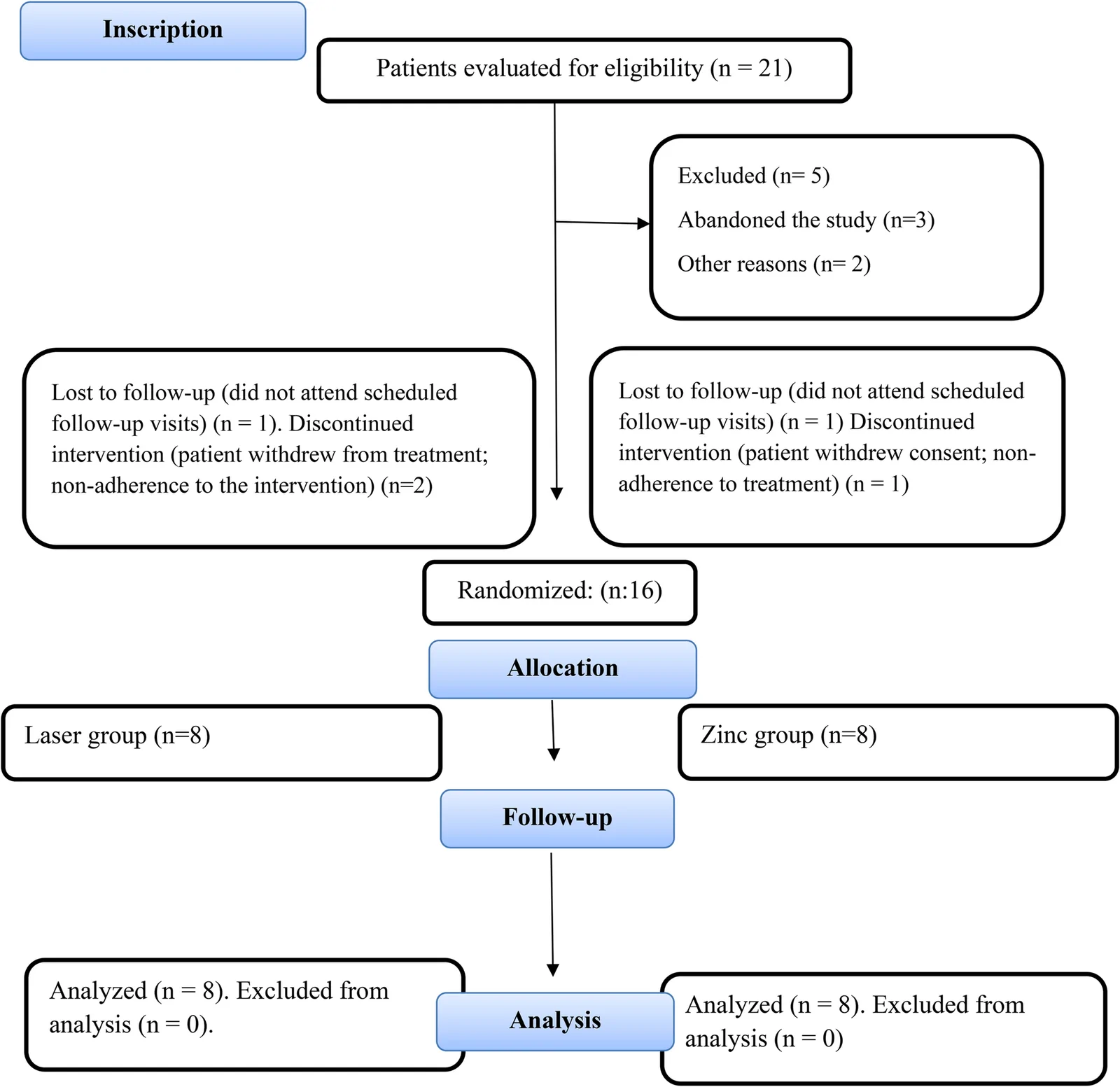
Flowchart based on CONSORT

Twenty-one patients were assessed for eligibility, of which 5 (3 abandoned the treatment, 1 completed treatment before the 30th radiotherapy session, and 1 did not adhere to the treatment) were excluded. A sample of 16 patients remained. These were randomly divided into a test group (Group A) and a control group (Group B), yielding 8 in each group (Fig. 1 - CONSORT Flowchart).

A simple randomization sequence was generated by the study supervisor using Microsoft Excel (Microsoft Corporation, Redmond, WA, USA) before the beginning of patient recruitment. The allocation sequence was stored in a password-protected spreadsheet and was not accessible to the investigators responsible for patient recruitment and treatment until allocation. Eligible participants were subsequently assigned to either the photobiomodulation or zinc sulfate group according to the pre-generated sequence.

Due to the distinct nature of the interventions, neither the participants nor the operator administering the treatments was blinded to treatment allocation. The same operator who administered the interventions also performed the objective taste assessments and was therefore not blinded to group allocation. However, the statistician was blinded to treatment identity during the statistical analysis and received the groups using coded labels.

Photobiomodulation therapy was performed in test group (A), using the Therapy XT laser (DMC^®^, São Carlos, SP, Brazil) with 100 mW of light output power at a continuous wavelength of 660 ± 10 nm (red) and 808 ± 10 nm (infrared). 2 J of red light was applied per point, (used for tissue regeneration), and 3 J of infrared light per point, which is used for paresthesia (respective energy density = 20.32 J/cm² and 30.48 J/cm²,). Treatment was initiated with the first radiotherapy session. Applications were made at 10 points on the dorsal and lateral regions of the tongue for 12 s at each point. Applications were performed twice a week until the end of radiotherapy treatment.

In the control group (B), zinc sulfate supplementation was administered orally in 45 mg capsules, three times daily after meals, from the first radiotherapy session until the end of treatment. Zinc sulfate was selected because it represented the standard institutional protocol for the management of treatment-related dysgeusia during the study period.

### Supportive oral care

In both groups, patients were treated for side effects that arose during antineoplastic treatment, such as mucositis, xerostomia, hyposalivation, and candidiasis. Oral hygiene measures were recommended for the management of mucositis, artificial saliva was used for the management of xerostomia and hyposalivation, and nystatin was prescribed for oral candidiasis when clinically indicated. These supportive interventions were provided equally to patients in both study groups and were not part of the randomized interventions.

### Objective taste test

In both groups, an objective taste identification test was performed at three time points: before the initiation of radiotherapy (T0), at the 15th radiotherapy session (T1), and at the 30th radiotherapy session (T2). For this, one hour before the test, patients were asked to stop eating, drinking, smoking, or using any oral hygiene products. Taste perception was evaluated using glucose (sweet), sodium chloride (salt), citric acid (sour), and urea (bitter), at a concentration of 1 mol/L for all tastes. Using pipette tips, one drop of each taste was placed in the center of the tongue and swallowed by the patient, starting with sweet, followed by bitter, sour, and salt, in sequence. Between each taste sample, the patient had 10 s to perceive and identify the taste. If the participant was unable to identify the taste, the procedure continued with the next taste modality. Between the different taste modalities, patients rinsed their mouths with distilled water. The tests were recorded in the patient’s clinical record.

The objective taste assessment protocol was adapted from the methodology previously described by Malta et al. in a randomized clinical trial that evaluated photobiomodulation for the prevention of chemotherapy-induced dysgeusia in patients with breast cancer. Four taste modalities (sweet, salty, sour, and bitter) were evaluated using standardized solutions prepared at a concentration of 1 mol/L. Umami was intentionally excluded because it was not included in the original protocol. The assessment focused on taste recognition rather than on the determination of taste detection thresholds. The protocol was standardized and applied uniformly to all participants throughout the study. Although the methodology was based on a previously published protocol, it was adapted to the clinical characteristics of patients undergoing radiotherapy for head and neck cancer and was not independently validated for this specific population [21].

### Statistical analysis

Because this was designed as a pilot study, no formal a priori sample size calculation was performed. Analyses were performed using complete cases. Statistical analyses were performed using IBM SPSS Statistics version 25.0 (IBM Corp., Armonk, NY, USA). All statistical tests were two-tailed, and statistical significance was established at *p* < 0.05.

For descriptive purposes, histological diagnosis was dichotomized as squamous cell carcinoma (SCC) or other histological types. Tumor location was classified as intraoral or extraoral, and tumor stage was categorized as early stage (T1–T2, N0, M0) or advanced stage (T3–T4 and/or N1–N3 and/or M1).

Categorical variables were expressed as absolute and relative frequencies, whereas continuous variables were summarized as mean ± standard deviation (SD) or median and interquartile range (IQR), according to data distribution. Normality was assessed using the Shapiro–Wilk test.

Baseline comparisons between the laser photobiomodulation and zinc supplementation groups were performed using Pearson’s chi-square test or Fisher’s exact test for categorical variables, as appropriate. Continuous variables with normal distribution were compared using the independent-samples Student’s t-test, whereas non-normally distributed variables were analyzed using the Mann–Whitney U test.

Taste perception was analyzed separately for each basic taste (sweet, bitter, sour, and salty). Between-group comparisons at each evaluation time point (baseline [T0], 15th radiotherapy session [T1], and 30th radiotherapy session [T2]) were performed using Pearson’s chi-square or Fisher’s exact test, as appropriate. For the 2 × 2 comparisons, odds ratios (ORs) with 95% confidence intervals (CIs) were additionally estimated to quantify the between-group effect, with the laser photobiomodulation group as the numerator and the zinc supplementation group as the reference. Given the small sample size and the presence of sparse and zero-frequency cells, ORs and 95% CIs were estimated using the conditional maximum likelihood method via the exact method associated with Fisher’s exact test. When the outcome was constant in both groups, the OR was considered not estimable.

Longitudinal changes in taste perception over time within each treatment group were assessed using Cochran’s Q test. When Cochran’s Q test was statistically significant, pairwise comparisons were conducted using McNemar’s test with Bonferroni correction for multiple comparisons. Accordingly, for the three planned pairwise comparisons, the statistical significance for post hoc analyses was established at *p* < 0.0167 (Bonferroni adjusted α = 0,05/3).

Given the exploratory nature of this pilot study and the very small sample size, longitudinal regression approaches such as generalized estimating equations or mixed-effects models were not employed because these methods rely on larger samples to provide stable parameter estimates and valid statistical inference. Instead, exact and non-parametric methods suitable for small samples were used [22–24].

## Results

Table 1 presents the clinical and demographic characteristics of the patients participating in the study. The sample consisted of 16 patients, with 8 in the Laser group and 8 in the Zinc group. The majority of participants were male (68.8%). The mean age was 65.6 ± 11.2 years, with a median of 66.5 years (IQR: 55.75–75).

**Table 1:** Clinical and demographic characteristics of the sample at baseline (T0)

Variable	Total (*N* = 16)	Laser (*N* = 8)	Zinc (*N* = 8)
Sex, n (%)			
Feminine	5 (31.3%)	4 (50.0%)	1 (12.5%)
Masculine	11 (68.8%)	4 (50.0%)	7 (87.5%)
Age (years),			
Average (SD)	65.63 (11.2)	62.75 (11.8)	68.5 (10.5)
Median (IQR)	66.5 (55.75-75)	62.5 (52.75–73.75)	70.5 (52.75-78)
Smoking, n (%)			
Non-smoker	6 (37.5%)	4 (50.0%)	2 (25.0%)
Former smoker	5 (31.3%)	3 (37.5%)	2 (25.0%)
Smoker	5 (31.3%)	1 (12.5%)	4 (50.0%)
Dichotomous smoking, n (%)			
Non-smoker + Ex-smoker	11 (68.8%)	7 (87.5%)	4 (50.0%)
Smoker	5 (31.3%)	1 (12.5%)	4 (50.0%)
Smoking time (years)*,			
Average (SD)	45.6 (9.7)	—	47 (10.6)
Median (IQR)	43.0 (37.5–55)		46.5 (37-57.5)
Cessation time (years)*			
Average (SD)	34 (6.5)	35 (5)	32.5 (10.6)
Median (IQR)	35.0 (27.5–40)	35.0 (—)	32.5 (—)
Histological type, n (%)			
CEC	15 (93.8%)	7 (87.5%)	8 (100%)
Others	1 (6.3%)	1 (12.5%)	0 (0%)
Tumor location, n (%)			
Intra-oral	5 (31.3%)	2 (25.0%)	3 (37.5%)
Extra-oral	11 (68.8%)	6 (75.0%)	5 (62.5%)
Staging, n (%)			
Home	7 (43.8%)	3 (37.5%)	4 (50.0%)
Advanced	9 (56.3%)	5 (62.5%)	4 (50.0%)
Chemotherapy, n (%)			
No	5 (31.3%)	2 (25.0%)	3 (37.5%)
Yes	11 (68.8%)	6 (75.0%)	5 (62.5%)
Dose per session (Gy)			
Average (SD)	2.06 (0.11)	2.06 (0.11)	2.06 (0.11)
Median (IQR)	2.0 (2-2.19)	2.0 (0)	2.0 (2-2.19)
Cumulative dose – 15th session (Gy),			
Average (SD)	30.04 (1.67)	30.94 (1.7)	30.94 (1.7)
Median (IQR)	30.0 (30-32.81)	30.0 (30-32.81)	30.0 (30-32.81)
Cumulative dose – 30th session (Gy),			
Average (SD)	61.88 (3.35)	61.88 (3.4)	61.88 (3.4)
Median (IQR)	60.0 (60-65.63)	60.0 (60-65.63)	60.0 (60-65.63)

^**x**^ Classification of numerical variables (based on normality tests)

Parametric variables

(*p* > 0.05 on Shapiro-Wilk)

• Age

• Duration of smoking

• Time since smoking cessation

Test: Independent samples t-test

Non-parametric variables

(*p* < 0.05 on Shapiro-Wilk)

• Dose per session

• Cumulative dose at the 15th session

• Cumulative dose at the 30th session

Test: Mann–Whitney U test

Regarding smoking status, 37.5% of the patients were non-smokers, 31.3% were former smokers, and 31.3% were current smokers. Considering the dichotomous variable, 68.8% were non-smokers or former smokers, and 31.3% were current smokers.

Among smokers and former smokers, the mean smoking duration was 45.6 ± 9.7 years, with a median of 43.0 years (IQR: 37.5–55), and the mean time to cessation was 34.0 ± 6.5 years, with a median of 35.0 years (IQR: 27.5–40).

The predominant histological type was squamous cell carcinoma (SCC), present in 93.8% of cases. Most tumors were located extra-orally (68.8%) and in an advanced stage (56.3%).

Regarding treatment, 68.8% of patients underwent chemotherapy combined with radiotherapy. The average dose per session was 2.06 ± 0.11 Gy. The average cumulative dose at the 15th session was 30.04 ± 1.67 Gy, and at the 30th session it was 61.88 ± 3.35 Gy.

Table 2 presents taste perception comparisons between groups over time points T0, T1, and T2. For sweet taste, at T2, the laser group experienced a greater decrease (only 50% could perceive it) compared to the zinc group (75%). For bitter taste, we noted a slight improvement in taste perception in the laser group, at T2, from 87.5% to 75% in the zinc group. In the sour taste test, we also observed a slight improvement in T2 in the laser group (87.5%) compared to the zinc group (62.5%). The salt taste appears to be the most affected in both groups at T1 and T2. In the zinc group, only 25% of patients-maintained perception at the end, compared to 50% in the laser group. Although we did not obtain a statistically significant difference, descriptive analyses suggested clinically relevant differences between the groups. Overall, no comparison between groups at T0, T1, or T2 was statistically significant (*p* > 0.05).

**Table 2:** Comparison of taste perception between the groups (Laser vs. Zinc) over time (T0, T1 and T2)

FLAVOR	TIME	LASER (*N* = 8)	ZINC (*N* = 8)		*p*-value		OR (95% CI)
Sweet	T0	8 (100%)	8 (100%)		—		NE
N	T1	8 (100%)	7 (87.5%)		1.000		∞ (0.026–∞)
	T2	4 (50.0%)	6 (75.0%)		0.608		0.358 (0.022–4.023)
Bitter	T0	8 (100%)	5 (62.5%)		0.200		0.358 (0.022–4.023)
	T1	5 (62.5%)	5 (62.5%)		1.000		1.000 (0.086–11.695)
	T2	7 (87.5%)	6 (75.0%)		1.000		2.214 (0.093–156.808)
Sour	T0	8 (100%)	8 (100%)		—		NE
	T1	5 (62.5%)	7 (87.5%)		0.569		0.260 (0.004–4.401)
	T2	7 (87.5%)	5 (62.5%)		0.569		3.839 (0.227–250.876)
Salt	T0	8 (100%)	6 (75.0%)		0.467		∞ (0.192–∞)
	T1	4 (50.0%)	2 (25.0%)		0.608		2.793 (0.249–45.763)
	T2	4 (50.0%)	2 (25.0%)		0.608		2.793 (0.249–45.763)

^x^ Data are presented as absolute frequency and percentage of participants correctly identifying each taste. ORs represent the odds of correct taste identification in the PBM group relative to the zinc sulfate group. Fisher’s exact test was used for between-group comparisons. *NE* not estimable. *OR not estimable because of a zero cell. For T0 sweet and sour, the outcome was constant in both groups and no association measure could be calculated

Between-group comparisons of taste perception at each evaluation time point are presented in Table 2. No statistically significant differences were observed between the laser photobiomodulation and zinc supplementation groups for any of the four basic tastes at baseline (T0), after the 15th radiotherapy session (T1), or after the 30th session (T2) (all *p* > 0.05). Although numerical differences were observed for sweet taste at T2 and salty taste at T1 and T2, these differences did not reach statistical significance.

Within-group longitudinal analyses are presented in Table 3. No statistically significant longitudinal changes were observed for bitter or sour taste in either treatment group (all Cochran’s Q *p* > 0.05). Significant overall changes were detected for sweet taste in the laser group (Q = 8.000, *p* = 0.018) and salty taste in the zinc group (Q = 8.000, *p* = 0.018). However, none of the pairwise comparisons evaluated by McNemar’s test remained statistically significant after Bonferroni correction (adjusted α = 0.0167). For some comparisons, McNemar’s test could not be estimated because one or more variables were constant or no discordant pairs were available.

**Table 3:** Within-group longitudinal changes in taste perception at baseline (T0), after the 15th radiotherapy session (T1), and after the 30th radiotherapy session (T2)

FLAVOR	GROUP	T0 *n*(%)	T1 *n*(%)	T2 *n*(%)	Q de Cochran (*p*)			Pairwise comparison (McNemar *p*-value)			Significant after Bonferroni correction (α < 0.0167)
					Q	gl	*p*-valor	T0 vs. T1	T0 vs. T2	T1 vs. T2	
SWEET	**Zinc**	8 (100%)	7 (87,5%)	6 (75,0%)	2,000	2	0,368	NE	NE	1	No
	**Laser**	8 (100%)	8 (100%)	4 (50,0%)	8,000	2	0,018	NE	NE	NE	No
BITTER	**Zinc**	5 (62,5%)	5 (62,5%)	6 (75,0%)	2,000	2	0,368	1	1	1	No
	**Laser**	8 (100%)	5 (62,5%)	7 (87,5%)	4,667	2	0,097	NE	NE	0,500	No
SOUR	**Zinc**	8 (100%)	7 (87,5%)	5 (62,5%)	4,667	2	0,097	NE	NE	0,500	No
	**Laser**	8 (100%)	5 (62,5%)	7 (87,5%)	3,500	2	0,174	NE	NE	0,625	No
SALT	**Zinc**	6 (75,0%)	2 (25,0%)	2 (25,0%)	8,000	2	0,018	0,125	0,125	NE	No
	**Laser**	8 (100%)	4 (50,0%)	4 (50,0%)	4,571	2	0,102	NE	NE	1	No

^x^: Data are presented as n (%). Within-group longitudinal differences were evaluated using Cochran's Q test. When applicable, pairwise comparisons were performed using the exact McNemar test. Bonferroni correction for three planned pairwise comparisons (T0 vs T1, T0 vs T2, and T1 vs T2) resulted in a significance threshold of α = 0.0167. NE = not estimable because one of the paired variables was constant or there were no discordant pairs. No = no pairwise comparison remained statistically significant after Bonferroni correction

## Discussion

The present randomized clinical trial evaluated the effect of photobiomodulation therapy on dysgeusia in patients undergoing radiotherapy for head and neck cancer. The main findings showed greater improvement in taste perception in the photobiomodulation group for salty, sour, and bitter tastes, particularly at T2, whereas sweet taste perception showed greater improvement in the zinc group. However, no statiscally significant differences were observed between the groups at any assessment time point. These results suggest that photobiomodulation may have potential as a supportive therapy for the management of treatment-related dysgeusia. Nevertheless, given the small sample size and considering the multifactorial nature of taste alterations and the influence of chemotherapy, tumor location, and patient-related factors, these findings should be interpreted with caution.

As shown by Silva et al. 2011, radiotherapy in doses ranging from 35 to 60 Gy combined with chemotherapy for head and neck cancer, was associated with alterations in taste perception for all evaluated taste modalities, with salt being the most affected and bitter the least. Gustatory function was tested with four substances: NaCl (Sodium Chloride), Sucrose, Citric Acid, and Urea in similar quantities and different concentrations. This hypothesis may explain the findings observed in the present study, in which salt taste was the most affected in both the laser and zinc groups, especially at T2 [10].

At T0 (before the start of radiotherapy) the zinc group already lacked some portion of their taste acuity, mainly the salt, sweet, and bitter tastes. MAES et al., [25], divided patients into radio treatment (RT) groups and evaluated at different times: pre-RT, 2 months post-RT, 6 months post-RT and 12–24 months post-RT. In the pre-RT group, a taste deficit was already respectively observed in patients for bitter (35%), salty (18%), and sweet (6%). The authors explained this pre-RT deficit as a consequence of tumor biology [26]. This assertion was corroborated in the present study, as patients in the zinc group with altered pre-radiotherapy (T0) taste perception presented primary tumors in the tongue region or adjacent sites. This deficit, noted before the start of radiotherapy can be explained by invasion of tumor cells towards the taste buds.

An improvement in taste perception was observed in the salt, sour, and bitter tastes in the laser group compared to the zinc group, mainly at T2. This result is consistent with previous studies, such as those by Camolesi et al. [28], Casco et al. [27], and Mobadder et al. [6]. Camolesi and colleagues observed in their study that the group receiving photobiomodulation presented a slower progression and lower degrees of dysgeusia (throughout the treatments) compared to the control group [28]. As previously highlighted, preventing taste disorders is essential for maintaining quality of life, particularly in patients undergoing radiotherapy for HNC [9, 21].

Even more advanced radiotherapy techniques result in some degree of toxicity to adjacent normal tissues. Thus, radiotherapy for head and neck cancer, even in cases where the treatment area is outside or adjacent to the oral cavity, can lead to taste dysfunction [9]. Nonetheless, advances in radiotherapy techniques, particularly intensity-modulated radiotherapy (IMRT) and helical IMRT, have improved the ability to spare organs at risk while maintaining adequate tumor coverage. These techniques reduce radiation exposure to the salivary glands and other healthy oral tissues and have consistently been associated with lower rates of radiation-induced conditions, which may contribute to improved quality of life. In addition, customized intraoral devices, including Cerrobend shielding stents and other patient-specific oral stents, may further reduce radiation exposure to adjacent healthy tissues. Although these approaches may help minimize treatment-related toxicities, evidence regarding their specific effect on radiation-induced dysgeusia remains limited and inconsistent, highlighting the need for further investigation [17, 29].

Silva et al. [10], conducted a quantitative and qualitative study in which taste function in patients with HNC undergoing radiotherapy inside and outside the oral cavity was evaluated. Fifty-six patients were included, and subjective and objective taste perception tests were performed before, immediately after, at 3 months, and at 6 months after radiotherapy treatment. In 29 patients, the irradiation field did not include the oral cavity whereas in 27 patients it did. Thus, it was found that patients with HNC undergoing radiotherapy presented alterations in taste; this, even when the irradiation does not encompass the oral cavity. The findings of the present study, where 68.8% of the tumors were located in the extra oral region and no significant differences were found between the sites, are consistent with their findings.

The management of treatment-related dysgeusia remains challenging, and interventions should be individualized according to patients’ symptoms and nutritional status. Preventive measures and supportive care are essential, including regular monitoring of taste alterations, adequate hydration, optimal oral hygiene, and tongue brushing, which may contribute to improved taste acuity. Patients may also benefit from dietary adaptations, such as consuming cold or lukewarm foods, frozen fruits, increasing seasoning or sweetness according to tolerance, choosing mild-flavored protein sources, avoiding foods with bitter or metallic tastes, reducing exposure to strong odors, and consuming smaller meals multiple times throughout the day. Nutritional assessment and personalized counseling are particularly important in patients with cancer-related taste disorders, as dysgeusia may negatively affect food intake, nutritional status, and quality of life. Zinc supplementation has been investigated as a therapeutic option due to its role in taste bud development and function. In the present study, zinc sulfate was selected as the comparison intervention due to its previous use in the management of taste alterations, although evidence regarding its clinical effectiveness remains inconclusive. However, its efficacy remains controversial, and excessive supplementation should be avoided due to potential adverse effects [30].

Other approaches, including saliva substitutes, nutritional supplements, pharmacological therapies, and physical interventions such as photobiomodulation, have been proposed, although further clinical studies are required to establish their effectiveness [30]. In the present study, photobiomodulation sessions were limited to twice weekly because of restricted access to the treatment facility. Although this frequency meets the minimum recommendation proposed by Gobbo et al. [4], the authors suggest that daily sessions during radiotherapy (5 days per week) may provide greater effectiveness in both preventive and therapeutic protocols.

The rationale for selecting zinc sulfate as the comparator should be considered when interpreting the present findings. Zinc supplementation was chosen because it represented the standard institutional protocol for the management of treatment-related dysgeusia during the study period. In addition, zinc has been widely investigated because of its role in taste bud development, maintenance, and regeneration. Therefore, the present study compared two active therapeutic interventions rather than comparing photobiomodulation with a placebo or no-treatment group. Consequently, the findings should be interpreted as a comparison between two clinically relevant treatment strategies rather than as evidence of the independent efficacy of photobiomodulation. Although photobiomodulation demonstrated greater improvements in some taste modalities, no statistically significant differences were observed between the groups.

This study has some limitations that should be considered when interpreting the findings. Due to logistical restrictions and limited access to the treatment facility, PBM was administered only twice weekly. Although this protocol met the minimum recommendations proposed by Gobbo et al. [4], preventive PBM protocols are more commonly performed on a daily basis during radiotherapy. Therefore, it is possible that the reduced frequency of PBM sessions may have underestimated the therapeutic potential of this intervention. Future studies should investigate whether daily PBM protocols could provide greater benefits in preventing or reducing treatment-related dysgeusia.

Another limitation concerns the taste assessment methodology. Although the protocol was adapted from a previously published study, it was designed to evaluate taste recognition rather than taste detection thresholds. Furthermore, the protocol was not independently validated for patients undergoing radiotherapy for head and neck cancer. Therefore, the results should be interpreted with caution, and future studies should consider the use of validated instruments capable of assessing both taste recognition and taste detection thresholds.

Another important limitation was the concomitant chemotherapy. Although participants undergoing radiotherapy were included according to the eligibility criteria, the timing of chemotherapy initiation was not standardized or incorporated into the study design. It is well known that antineoplastic agents used in chemotherapy are toxic to rapidly proliferating normal tissues, including those of the oral cavity, and can cause direct damage to taste bud cells or indirectly affect brain regions involved in taste perception. Since chemotherapy-related taste alterations may occur within the first few days after treatment initiation, differences in the timing of chemotherapy among participants may have influenced the severity of dysgeusia and, consequently, the treatment outcomes. In addition, 68.8% of the participants underwent concomitant chemoradiotherapy, a treatment combination known to exert a synergistic detrimental effect on taste perception, which may have further contributed to variability in the observed outcomes [21].

Another factor that must be considered is smoking. Although smoking status was collected, smoking status was not considered as a stratification factor during randomization. Approximately one-third of the participants were smokers, and tobacco use may independently affect taste perception, potentially influencing the assessment of treatment-related dysgeusia [10]. Given the small sample size, simple randomization may not have ensured a balanced distribution of smokers between the treatment groups, and therefore, a potential residual confounding effect of smoking cannot be excluded. Although baseline differences were not statistically significant, a higher proportion of current smokers was observed in the zinc group, which may have influenced taste perception. Future randomized clinical trials should consider stratified randomization according to smoking status and chemotherapy exposure or timing to minimize the influence of these potential confounders and strengthen the internal validity of the findings.

Supportive care for treatment-related oral complications represents another potential confounding factor. Although both groups received the same supportive oral care, including oral hygiene measures for mucositis, artificial saliva for xerostomia, and nystatin for oral candidiasis when clinically indicated, these conditions and their respective treatments may influence taste perception. Mucositis, salivary dysfunction, and oral candidiasis may independently affect gustatory function, while some supportive treatments may also modify the oral environment and taste perception. Therefore, their potential contribution to the observed changes in dysgeusia cannot be completely excluded. Nevertheless, because the same supportive care was provided to both groups, differential effects attributable to supportive treatment are expected to have been minimized.

Another limitation of this study was the absence of blinding of participants, the treatment operator, and the outcome assessor. Because the photobiomodulation and zinc sulfate interventions differed substantially in their mode of administration, blinding of participants and the operator was not feasible. Furthermore, the same operator who administered the interventions performed the objective taste assessments and was aware of treatment allocation, which may have introduced a risk of assessment bias. Although the statistician was blinded to treatment identity, this measure does not eliminate the potential influence of non-blinded outcome assessment. Future randomized clinical trials should incorporate blinded outcome assessment whenever feasible to reduce the risk of bias.

Because of the limited sample size, this study was underpowered to detect small or moderate differences between treatments. Therefore, the absence of statistically significant differences should not be interpreted as evidence of equivalence between interventions. Based on our findings, photobiomodulation may represent a promising therapeutic approach for the management of treatment-related dysgeusia. However, larger randomized clinical trials are necessary to determine its efficacy.

## Conclusion

The results of this study indicate that photobiomodulation (PBM) has the potential to reduce the severity of dysgeusia associated with head and neck cancer treatment particularly during the most critical phases of treatment. However, these data should be interpreted with caution, since no statistically significant differences were obtained between the groups, emphasizing the importance of conducting new randomized controlled trials with larger samples and adequate statistical power.

## Data Availability

No datasets were generated or analysed during the current study.

## Declarations

### Clinical trial registration details

This study was prospectively registered in the Brazilian Registry of Clinical Trials (ReBEC) under registration number RBR-2tkmcx6. Registration date: February 3, 2025.
